# Dissecting Transient Burst Events

**DOI:** 10.1016/j.tics.2020.07.004

**Published:** 2020-10

**Authors:** Catharina Zich, Andrew J. Quinn, Lydia C. Mardell, Nick S. Ward, Sven Bestmann

**Affiliations:** 1 Department of Clinical and Movement Neuroscience, UCL Queen Square Institute of Neurology, London, UK; 2 Wellcome Centre for Integrative Neuroimaging, FMRIB, Nuffield Department of Clinical Neurosciences, University of Oxford, Oxford, UK; 3 Oxford Centre for Human Brain Activity, Wellcome Centre for Integrative Neuroimaging, Department of Psychiatry, University of Oxford, Oxford, UK; 4 Wellcome Centre for Human Neuroimaging, UCL Queen Square Institute of Neurology, London, UK

**Keywords:** burst events, oscillations, multidimensional

## Abstract

Increasing efforts are being made to understand the role of intermittent, transient, high-power burst events of neural activity. These events have a temporal, spectral, and spatial domain. Here, we argue that considering all three domains is crucial to fully reveal the functional relevance of these events in health and disease.

Neural activity recorded from the scalp [magneto-/electroencephalogram (M/EEG)], cortical surface [electrocorticogram (EcoG)], or inside the brain [local field potential (LFP)] is traditionally analysed by averaging tens to hundreds of trials. The trial-wise average of spectrograms commonly shows periods of sustained high or low spectral power. The underlying single trial activity may manifest as oscillations (i.e., sustained rhythmic fluctuations of synchronous spiking activity) with periods of high or low amplitude. However, as recently demonstrated, sustained high spectral power in the averaged spectrogram can also arise from the accumulation of burst-like events across trial [1., 2., 3., 4., 5., 6.]. Burst-like events are intermittent, transient periods of synchronous spiking activity, the generator of which may or may not be rhythmic [7]. Accordingly, the underlying mechanism of high or low spectral power in the averaged spectrogram can be due to differences in event amplitude, or other event characteristics. Describing events provides an untapped opportunity to expand our understanding of brain function in health and disease.

## Domain Reduction to Characterise Events

**Figure 1:**
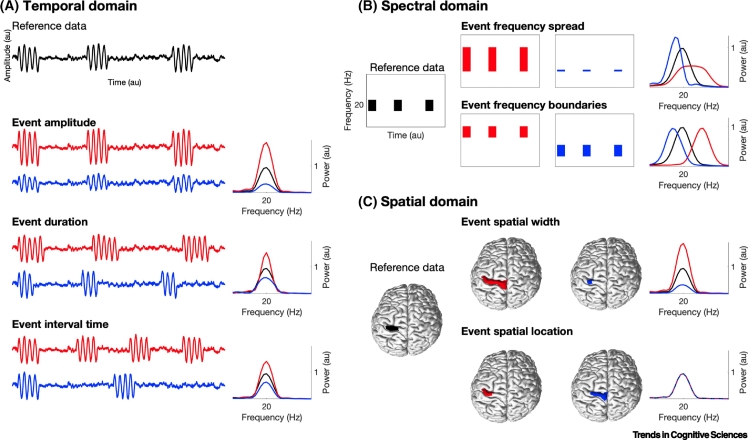
Main Temporal, Spectral, and Spatial Event Characteristics. Events can be characterised in the temporal, spectral, and spatial domains. For each domain, the main event characteristics are presented. Each event characteristic is illustrated using two exemplary data (red and blue) relative to a reference data (black) and their derived static spectral power estimates. (A) Temporal domain: event amplitude (traditionally seen as a temporal event characteristic), event duration, and event interval time. (B) Spectral domain: frequency spread and frequency boundaries of the event. (C) Spatial domain: spatial width and spatial location of the event. As evident, the mechanism underlying differences in spectral power can be manifold within and across domains. As an example, an increase in spectral power can be caused by larger event amplitude, longer event duration, shorter event interval time, narrower event frequency spread, and larger event spatial width. Furthermore, the characteristics depicted in (A–C) can also interact, or can be conditionally dependent within and/or across domains (for details on domain interactions, see Figure 2 in the main text).

Events have a temporal, spectral, and spatial domain. However, most work so far has focussed on temporal event characteristics, such as event amplitude (traditionally seen as a temporal event characteristic), event duration, or event interval time (Figure 1A). Events are thereby compressed to a singular dimension, by reducing the spatial and spectral domains of their underlying signals. However, this domain reduction removes potentially relevant aspects of the data.

The spatial domain is commonly reduced by extracting the time series from a single spatial location, or summarising the time series of several spatial locations through their average or a linear combination [e.g., first principle component (PC)]. Similarly, the spectral domain is often reduced by selecting a single peak frequency, averaging the signal or amplitude envelope within a specified frequency band, or by fitting a state-wise frequency profile from a time-delay embedded or autoregressive-based Hidden Markov Model (HMM) [8].

Rarely, both the temporal and spectral characteristics of events are analysed together (Figure 1B [1]), but the spatial characteristics (Figure 1C) have not yet been incorporated. Given that differences in spectral power can be caused by changes in a variety of event characteristics (e.g., event duration and/or increase in event spatial width; Figure 1), consideration of all three domains is likely to be necessary for disclosing the underlying mechanisms of differences in population activity measures, such as spectral power.

## Domain Reduction Can Deceive

**Figure 2:**
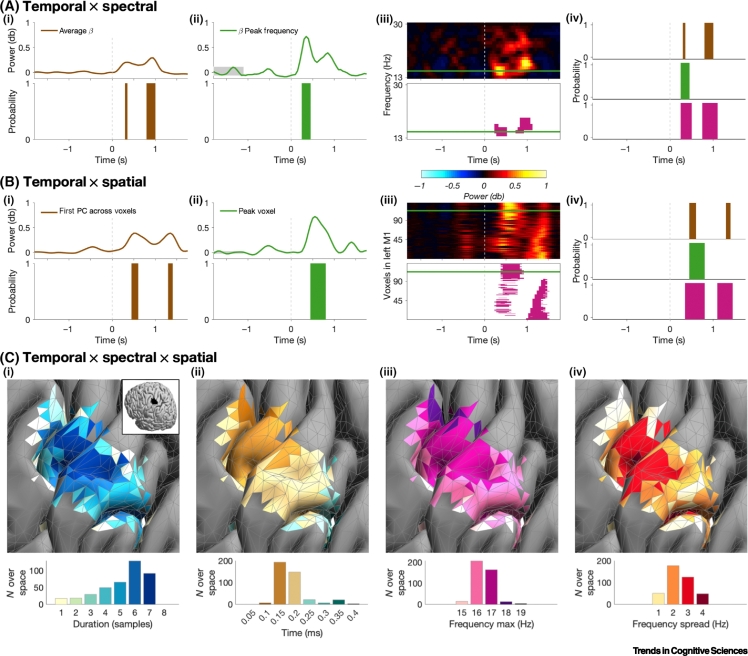
Problems of Domain Reduction and Opportunities of the Multidomain Approach.

Domain reduction can have several unwanted consequences. First, different domain reduction methods likely result in differences in event characteristics. Focussing on the temporal domain, Figure 2A,B illustrates how event duration, event interval time, and event onset differ across common domain reduction approaches. Importantly, these differences are not attributable to differences in how events are identified (e.g., threshold).

Furthermore, domain reduction can obscure interactions between domains, which may lead to ambiguous inferences and conclusions drawn from these analyses. For example, if events change their location in the spatial and/or spectral domain over the course of the event, domain reduction can lead to misestimation of the event duration. The second event in Figure 2B illustrates this point. As evident from Figure 2Biii*,* the event has a temporospatial gradient. Therefore, reducing the spatial domain can result in an underestimation of the actual event duration or missing the event altogether.

Thus, it becomes apparent that domain reduction can hide important information about mass neural signals. Therefore, even when only one domain is of interest, considering all domains is most likely necessary to accurately describe each individual domain. This increases sensitivity to detect differences between conditions, individuals, or groups, and to minimise spurious inference.

## Characterising Events: A Multidimensional Problem

Considering all domains simultaneously yields a more detailed description of the underlying signal and allows interactions across domains to be characterised. These interactions can have many forms, and here we illustrate four such interactions between domains (Figure 2C) with a more detailed discussion of the interactions depicted in Figure 2Cii,iii.

As shown in Figure 2Cii,iii, the spatial location of the event shifts from medial to lateral over the course of the event, while at the same time the upper frequency boundary of the event shifts to a lower frequency. Such a temporal–spectral–spatial interaction appears plausible given previous work outside the burst literature. For example, temporal–spatial interactions (travelling waves) have been described at similar temporal and spatial scales as burst events [9], and sensorimotor beta activity travels in a consistent anatomical direction [10]. As another example, β peak frequency varies within different locations of the sensorimotor network (i.e., spectral–spatial interaction [11]).

These examples support the idea of interactions across the three domains. They illustrate how multidomain analysis can reveal gradients that would otherwise remain obscure but that are relevant for revealing the mechanistic role of events.

## Prospects, Challenges, and Concluding Remarks

Analysis of all three domains (with their five dimensions: temporal–spectral–spatial [x–y–z]) benefits from high signal-to-noise recordings of neural activity, which have become more readily accessible through recent advances in acquisition techniques of mass neural activity, such as large-array, high-density ECoG, high-precision MEG, or optically pumped magnetometers. Parallel development in signal processing, particularly effective spatial leakage correction, precise beamformer, high-resolution time-frequency analysis, and nonstationary approaches for event identification constitute additional advances for analysing events precisely across domains. These advances notwithstanding, challenges, such as varying signal-to-noise across time points, frequencies, and spatial locations, tools for analysing multidimensional event characteristics effectively across events and individuals, and, for M/EEG data, accuracy of spatial localisation (forward and inverse modelling), remain. Furthermore, the resolution of the temporal and spectral domains constitutes a compromise and depends on the choice of ‘epoch’ length in the time–frequency analysis.

In summary, intermittent, transient, events have a temporal, spectral, and spatial domain. Considering all three domains will yield a more accurate description of individual event characteristics and their interactions. This is likely to underpin identification of more precise fingerprints of mechanisms underlying differences in population activity measures, such as spectral power. In turn, this can guide the development of rational and mechanistically grounded treatments that target neurological and psychiatric conditions. While we use sensorimotor β activity here as a showcase for discussing the impact of domain reduction and opportunities to consider all three domains, these points likely extend to other brain areas, frequency ranges, and pathologies.
